# Si-Wu-Tang Alleviates Nonalcoholic Fatty Liver Disease via Blocking TLR4-JNK and Caspase-8-GSDMD Signaling Pathways

**DOI:** 10.1155/2020/8786424

**Published:** 2020-08-11

**Authors:** Yaxing Zhang, Ge Zhou, Zifeng Chen, Weibing Guan, Jiongshan Zhang, Mingmin Bi, Fenglin Wang, Xinchao You, Yangjing Liao, Shuhui Zheng, Kangquan Xu, Hongzhi Yang

**Affiliations:** ^1^Department of Traditional Chinese Medicine, The Third Affiliated Hospital, Sun Yat-sen University, Guangzhou, China; ^2^Institute of Integrated Traditional Chinese and Western Medicine, Sun Yat-sen University, Guangzhou, China; ^3^Biofeedback Laboratory, Xinhua College of Sun Yat-sen University, Guangzhou, China; ^4^School of Biomedical Engineering, Xinhua College of Sun Yat-sen University, Guangzhou, China; ^5^Department of Otorhinolaryngology, The Seventh Affiliated Hospital, Sun Yat-sen University, Shenzhen, China; ^6^Zhongshan School of Medicine, Sun Yat-sen University, Guangzhou, China; ^7^Department of Science and Education, The First Affiliated Hospital/School of Clinical Medicine of Guangdong Pharmaceutical University, Guangzhou, China; ^8^Research Center for Translational Medicine, The First Affiliated Hospital, Sun Yat-sen University, Guangzhou, China

## Abstract

**Background:**

Nonalcoholic fatty liver disease (NAFLD) has high global prevalence; however, the treatments of NAFLD are limited due to lack of approved drugs.

**Methods:**

Mice were randomly assigned into three groups: Control group, NAFLD group, NAFLD plus Si-Wu-Tang group. A NAFLD mice model was established by feeding with a methionine- and choline-deficient (MCD) diet for four weeks. Si-Wu-Tang was given orally by gastric gavage at the beginning of 3rd week, and it lasted for two weeks. The treatment effects of Si-Wu-Tang were confirmed by examining the change of body weight, serum alanine aminotransferase (ALT) and aspartate transaminase (AST) levels, Oil Red O staining, and hematoxylin and eosin (H&E) staining of the liver samples and accompanied by steatosis grade scores. The expression and activation of the possible signaling proteins involved in the pathogenesis of NAFLD were determined by western blotting.

**Results:**

Mice fed with four weeks of MCD diet displayed elevated serum levels of ALT and AST, while there was decreased body weight. The hepatic Oil Red O staining and H&E staining showed severe liver steatosis with high steatosis grade scores. All these can be improved by treating with Si-Wu-Tang for two weeks. Mechanistically, the increased hepatic TLR4 expression and its downstream JNK phosphorylation induced by MCD diet were suppressed by Si-Wu-Tang. Moreover, the upregulations of Caspase-8, gasdermin D (GSDMD), and cleaved-GSDMD in liver mediated by MCD diet were all inhibited by Si-Wu-Tang.

**Conclusions:**

Treatment with Si-Wu-Tang improves MCD diet-induced NAFLD in part via blocking TLR4-JNK and Caspase-8-GSDMD signaling pathways, suggesting that Si-Wu-Tang has potential for clinical application in treating NAFLD.

## 1. Background

Nonalcoholic fatty liver disease (NAFLD) is defined as the presence of >5% hepatic steatosis (either by histology or imaging techniques); there must be lack of secondary causes of hepatic fat accumulation, such as significant alcohol consumption, long-term use of a steatogenic medication, or monogenic hereditary disorders [1–3]. NAFLD encompasses a broad spectrum of conditions, from simple steatosis (referred to as nonalcoholic fatty liver (NAFL)), through nonalcoholic steatohepatitis (NASH), to fibrosis, and ultimately cirrhosis and hepatocellular carcinoma (HCC) [4, 5]. Moreover, NAFLD is associated with higher risks of severe extrahepatic diseases, such as cardiovascular diseases [1–3]. The global prevalence of NAFLD is estimated to be approximately 24-25%, and the pooled overall nationwide prevalence of NAFLD is estimated to be 29.2% in China [2, 6, 7]. Therefore, it is urgent to discover novel strategies for treating NAFLD.

A number of traditional Chinese medicinal formulas provide the promising sources to develop alternative and complimentary medicines for NAFLD therapy and prevention [8–11]. Si-Wu-Tang, a traditional Chinese medicinal formula, including *Rehmanniae Radix* (Shu Di Huang), *Angelica Radix* (Dang Gui), *Paeoniae Radix* (Bai Shao Yao), and *Chuanxiong Rhizoma* (Chuan Xiong), has been shown to improve the antioxidant level and positively regulate the lipid profile, liver function, and skin integrity and texture in healthy adults [12]. This traditional Chinese medicinal formula was first recorded in *Xian Shou Li Shang Xu Duan Mi Fang* (《仙授理伤续断秘方》) by *Lin Daoren* in China Tang Dynasty. Si-Wu-Tang has been traditionally used for treating gynecological diseases, such as relief of menstrual irregularity, dysmenorrhea, uterine bleeding, climacteric syndrome, and other estrogen-related diseases, since it is recorded in the official Chinese medicine classics *Tai Ping Hui Min He Ji Ju Fang* (《太平惠民和剂局方》) in China Song Dynasty [13–20].

By the basic theory of Traditional Chinese Medicine, Si-Wu-Tang is a blood-building decoction (Chinese Medical Concept: Bu-Xue/补血) to improve a deficiency of blood (血虚) [13, 21–23]. The animal studies indicated that Si-Wu-Tang has strong abilities to improve blood deficiency induced by radiation [21, 24, 25], or cyclosphosphamide [26], or the compound methods of bleeding, starved feeding, and exhausting [27]. Mechanistically, Si-Wu-Tang improved hematopoietic function of bone marrow by modulating apoptosis, proliferation, and differentiation-related genes expression in haematopoietic stem/progenitor cell [26], alleviated disorders of carbohydrate and lipid metabolisms, disorders of immune function, and the damage of mitochondria and lymphocyte observed in blood deficiency animal models [21, 28, 29]. Si-Wu-Tang administration before irradiation reduced the frequency of radiation-induced apoptosis in crypt of intestine [22] and alleviated intestinal inflammatory processes and protected against intestinal mucosa injury [25]. However, its effects on NAFLD are not clear.

The changes in diet, gut microbiome and the sedentary lifestyle-associated behavior, and the genetic or epigenetic backgrounds that determine relative susceptibility to NAFLD, lead to increased metabolic substrate delivery to the liver and activation of systemic inflammatory changes, causing insulin resistance [30, 31]. These changes drive increased circulating inflammatory cytokines to induce hepatocellular oxidative stress or endoplasmic reticulum stress and modify cell-cell crosstalk, resulting in cell injury or death, inflammation, fibrogenesis and genomic instability that predispose to cirrhosis and HCC [4, 30, 31]. Therefore, inflammation, insulin resistance, oxidative stress, and cell death are implicated in the pathogenesis of NAFLD. Among the above pathogenesis, TLR4-mediated innate immune signaling plays an essential role in the development of NAFLD. TLR4 is involved in the pathogenesis of fructose-induced, high-fat and high-cholesterol diet-induced, or methionine- and choline-deficient (MCD) diet-induced hepatic steatosis in mice [32–35]. Recent studies indicated that liver localization of lipopolysaccharides (LPS), which is the ligand of TLR4, is increased in both human and the experimental NAFLD mice model [36]. Mechanistically, TLR4-induced JNK phosphorylation and their downstream Caspase-8 activation contribute to the pathogenesis of NAFLD [37–49]. Therefore, hepatocyte-specific Caspase-8 knockout ameliorates the development of MCD diet-induced NASH by modulating liver injury and attenuates alcoholic hepatic steatosis in mice [39, 50]. Moreover, Caspase-8 activation can lead to the cleavage of gasdermin D (GSDMD) [51–53]. Cleaved-GSDMD forms membrane pores that lead to cytokine release and/or programmed lytic cell death, called pyroptosis [54]. GSDMD plays a key role as a pyroptosis executor in the pathogenesis of NASH by regulating lipogenesis, promoting proinflammatory cytokines secretion, exacerbating NF-ĸB activation, thus directly or indirectly facilitating liver fibrosis and lipogenesis [55]. Therefore, these innate immune signaling pathways are the essential drug targets for treating NAFLD.

Si-Wu-Tang has strong anti-inflammatory and antioxidative effects [12, 13, 17, 56–60]; it can improve carbohydrate and lipid metabolisms in blood deficiency animal models [21, 28, 29] and positively regulate the lipid profile and liver function in healthy adults [12]. Based on these, we speculated that Si-Wu-Tang might have therapeutic effect on NAFLD. If exists, we will further investigate whether the novel inflammatory and cell death mechanisms of NAFLD discussed above are involved in its protective effects.

## 2. Methods

### 2.1. Preparation of Si-Wu-Tang

The drug composition of Si-Wu-Tang (41 g) was according to *Tai Ping Hui Min He Ji Ju Fang* (the official Chinese medicine classics in China Song Dynasty) and the recent literatures [14, 17, 20, 26, 28, 29, 61, 62] (Table 1). The four plant materials prepared in ready-to-use forms were bought from DaShenLin Pharmaceutical Group Co., Ltd. (Guangzhou, China). They were identified according to the first volume of the *Chinese Pharmacopoeia 2015 edition* by Wang Yonggang, an associate professor in Guangdong Engineering and Technology Research Center for Quality and Efficacy Reevaluation of Post-Market Traditional Chinese Medicine, Sun Yat-sen University (Guangzhou, China). The voucher specimens of Shu Di Huang (no. 2019-BL-016), Dang Gui (no. 2019-BL-017), Bai Shao Yao (no. 2019-BL-018), and Chuan Xiong (no. 2019-BL-019) were deposited in Biofeedback Laboratory, Xinhua College of Sun Yat-sen University (Guangzhou, China).

The quality standards of the four plant materials of Si-Wu-Tang conformed to the *Chinese Pharmacopoeia 2015 edition*. The prescription of Si-Wu-Tang was based on the clinical application method in Department of Traditional Chinese Medicine, The Third Affiliated Hospital, Sun Yat-sen University (Guangzhou, Guangdong, China), and the previously described methods [14, 20, 26, 28, 29, 61–63]. Briefly, the dried prescriptions of Si-Wu-Tang (2 × , 82 g) were soaked in 200 ml distilled water for 30 minutes. Then, they were decocted twice with boiling water in a pot made of purple clay with an automatic liquid heater (HuFu Hardware Factory, Chaozhou, Guangdong, China), and they were condensed to 82 ml. Thus, the concentration of Si-Wu-Tang was 1 g/ml (dried herb weight/solution), which was filtered and stored at 4°C before use [20, 29]. Si-Wu-Tang solution was prepared every 3 days.

### 2.2. The Methionine- and Choline-Deficient (MCD) Diet-Induced NAFLD Animal Model and Treatment Protocol

Male C57BL/6J mice were purchased from Guangdong Medical Laboratory Animal Center (Foshan, Guangdong, China). Mice aged about 8–10 weeks were used in this study. All animals were housed in a temperature-controlled animal facility with a 12-hour light-dark cycle and allowed to obtain rodent chow and water *ad libitum.* All animals received humane care, and all animal procedures were approved by the Institutional Animal Care and Use Committee of Sun Yat-sen University (no. 2018-057), and these conformed to the Principles of Laboratory Animal Care formulated by the National Institutes of Health guide for the care and use of Laboratory animals (NIH Publications no. 8023, revised 1978) [64].

Mice were randomly assigned to three groups: Control group (*n* = 10), MCD group (*n* = 10), and MCD plus Si-Wu-Tang group (*n* = 10). As previously revealed [65], mice in MCD group and MCD plus Si-Wu-Tang group were *ad libitum* fed with a methionine- and choline-deficient diet (MCD; MD12052, Mediscience Ltd., Yangzhou, China) for four weeks to induce NAFLD. Mice in Control group were *ad libitum* fed with an identical diet sufficient in methionine and choline (MCD control; MD12051, Mediscience Ltd., Yangzhou, China) for four weeks. The histologic evidence shown that there was significant hepatic steatosis in mice fed with the MCD diet for 7, 10, or 14 days [66–70]. Therefore, in order to investigate whether Si-Wu-Tang has the therapeutic effect on MCD diet-induced NAFLD, mice in MCD plus Si-Wu-Tang group were daily given Si-Wu-Tang from the 15th day at 16:00-17:00 for two weeks. As previously described [20, 28, 29, 61, 62], mice received Si-Wu-Tang at the dose of 1 ml per 100 g body weight (1 g/100 g body weight) orally by gastric gavage (without fasting). On 29th day, the mice were fasted for four hours before sample collection.

### 2.3. Measurement of Serum ALT and AST Concentrations

Blood samples were collected by cardiac puncture after the mice were euthanized. Samples were allowed to sit for 30 minutes at room temperature for coagulation [71]. Then, they were centrifuged at 3000 rpm at 4°C for 10 minutes, and the supernatant serum was collected and stored at −80°C until analysis [71]. The serum levels of alanine aminotransferase (ALT) and aspartate transaminase (AST) were analyzed by an automatic blood chemistry analyzer (HITACHI 7600, Tokyo, Japan) in the Department of Clinical Laboratory, The Third Affiliated Hospital, Sun Yat-sen University.

### 2.4. Histopathology Analysis

After collecting blood samples, livers were harvested for observing histological alterations by hematoxylin and eosin (H&E) staining and Oil Red O staining as previously revealed [65, 72]. Briefly, liver sections embedded in paraffin were stained with H&E, and Oil Red O (Sigma, #O0625) staining was performed in frozen liver sections prepared in Tissue-Tek® optimum cutting temperature (O.C.T.) compound (Sakura, #4583). H&E sections were graded for hepatic steatosis as previously described [73, 74]. Briefly, steatosis was scored and the severity was graded, based on the percentage of the total area affected, into the following categories: 0 (<5%), 1 (5–33%), 2 (>33–66%), and 3 (>66%) [73, 74].

### 2.5. Western Blotting

The antibody against TLR4 (#sc-293072) was from Santa Cruz Biotechnology (Santa Cruz, CA, USA). The antibodies against c-Jun NH_2_-terminal kinase (JNK) (#9252S), p-JNK (#9255S) and Caspase-8 (#9746S), anti-rabbit IgG HRP-linked antibody (#7074S), and anti-mouse IgG HRP-linked antibody (#7076S) were from Cell Signaling Technology (Danvers, MA, USA). The antibody against GSDMD (#ab219800) was from Abcam (Cambridge, UK). Glyceraldehyde-3-phosphate dehydrogenase (GAPDH) antibody (#MB001) was from Bioworld Technology (Qixia District, Nanjing, China).

Western blotting was performed as we have previously described [64]. The proteins were transferred to polyvinylidene fluoride membranes (Millipore, Bedford, MA, USA), which were incubated with primary and secondary antibodies by standard techniques. The enhanced chemiluminescence (ChemiDoc XRS + System, Bio-Rad, Hercules, CA, USA) was used to accomplish immunodetection.

### 2.6. Statistical Analysis

Data were expressed as mean ± SD. Statistical analyses were performed by one-way analysis of variance (ANOVA) followed by Bonferroni's post-hoc test or by Kruskal–Wallis test followed by Dunn's post-hoc test. A value of *p* < 0.05 was considered as significantly different. All statistical analyses were performed using GraphPad Prism 5.0.

## 3. Results

### 3.1. The Effects of Si-Wu-Tang on Body Weight in MCD Diet-Fed Mice

It has been well known that MCD diet can induce body weight loss in mice [75]. Therefore, we measured the body weight of mice at the beginning and at the end of the experiment. As shown in Table 2, there was no difference in the body weight of mice from these three groups at the beginning. After feeding with four weeks of MCD diet, the body weight decreased significantly in MCD group (*p* < 0.001 vs. Control) and in MCD plus Si-Wu-Tang group (*p* < 0.001 vs. Control). Moreover, two weeks of Si-Wu-Tang treatment improved the loss of body weight (*p* < 0.001 vs. MCD) (Table 2).

### 3.2. Si-Wu-Tang Improved Liver Damage in Mice Fed with a MCD Diet

**Figure 1:**
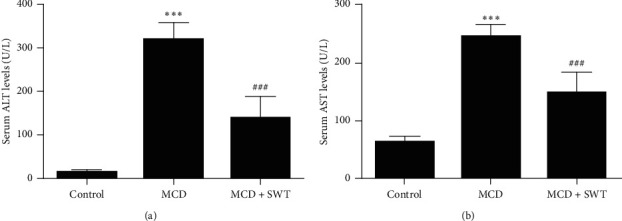
Si-Wu-Tang decreased MCD diet-induced elevated serum ALT and AST levels. (a) Serum ALT levels in three groups. (b) Serum AST levels in three groups. SWT, Si-Wu-Tang; ALT, alanine aminotransferase; AST, aspartate transaminase; *n* = 10, ^*∗∗∗*^*p* < 0.001 vs. Control; ^###^*p* < 0.001 vs. Control or MCD.

In order to investigate whether Si-Wu-Tang has the protective effects on liver damage induced by MCD diet, we determined the serum levels of ALT and AST, which are markers of liver damage. Compared with Control group, mice fed with a MCD diet for four weeks resulted in a major increase in serum ALT (319.2 ± 39.4 U/L vs. 13.8 ± 5.7 U/L, *p* < 0.001) and AST (247.2 ± 20.2 U/L vs. 62.4 ± 10.3 U/L, *p* < 0.001) levels (Figures 1(a) and 1(b)). Two weeks of Si-Wu-Tang treatment decreased serum ALT (139.2 ± 48.6 U/L vs. 319.2 ± 39.4 U/L, *p* < 0.001) and AST (149.4 ± 35.3 U/L vs. 247.2 ± 20.2 U/L, *p* < 0.001) levels when compared with MCD group (Figures 1(a) and 1(b)). However, the ALT and AST levels in MCD plus Si-Wu-Tang group were still higher than those in Control group (*p* < 0.001) (Figures 1(a) and 1(b)). Therefore, the observation that the serum levels of ALT and AST in MCD plus Si-Wu-Tang group were lower than those in the MCD group indicate a protective effect of Si-Wu-Tang against liver damage.

### 3.3. Si-Wu-Tang Alleviated Liver Steatosis in Mice Fed with a MCD Diet

**Figure 2:**
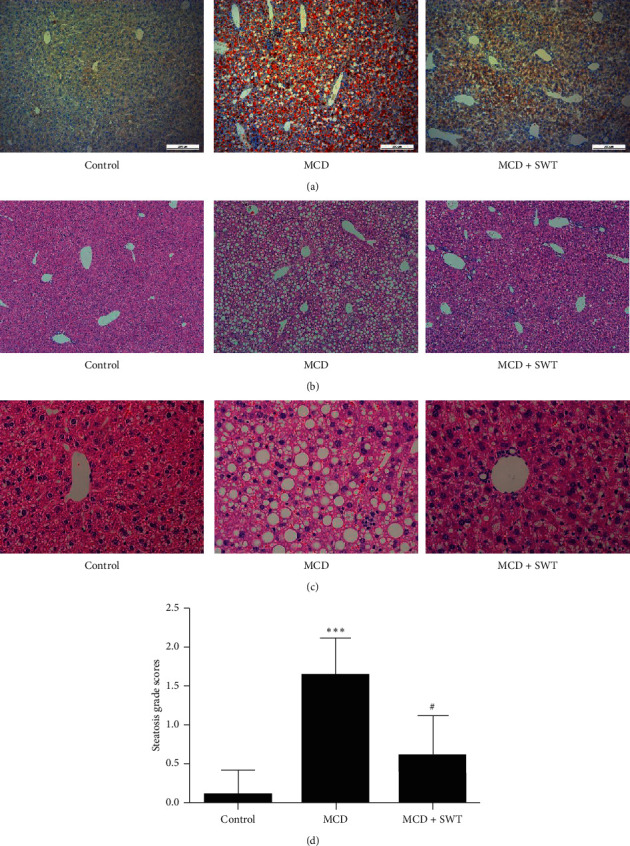
Si-Wu-Tang improved MCD diet-induced liver steatosis. Liver histology was evaluated by Oil Red O staining (a), H&E staining ((b) 100^×^; (c) 400^×^), and the steatosis grade scores (d) in three groups. SWT, Si-Wu-Tang; *n* = 10, ^*∗∗∗*^*p* < 0.001 vs. Control; ^#^*p* < 0.05 vs. MCD.

Similar to that in humans with NASH, the characteristic pathology of MCD diet-fed mice revealed macrovesicular lipid accumulation [76, 77]. Oil Red O staining displayed that MCD diet induced severe lipid droplet accumulation in the liver, which was alleviated by Si-Wu-Tang treatment (Figure 2(a)). Similarly, H&E staining showed that there were hepatic lipid accumulation as clear macrovacuoles, and the increased steatosis grade scores indicated that MCD diet induced severe liver steatosis; all these were improved by treatment with two weeks of Si-Wu-Tang (Figures 2(b)–2(d)). Thus, Si-Wu-Tang improved liver steatosis in mice fed with a MCD diet.

### 3.4. Si-Wu-Tang Inhibited Hepatic TLR4-JNK Signaling in Mice Fed with a MCD Diet

**Figure 3:**
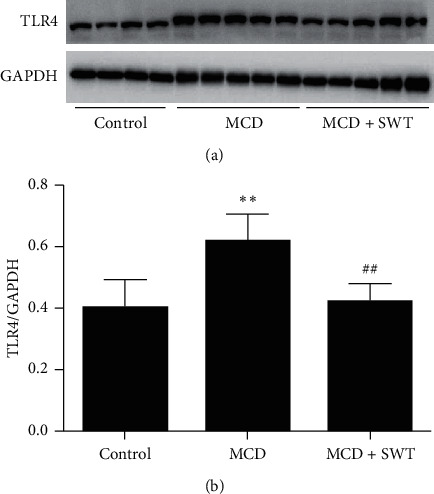
Si-Wu-Tang reduced MCD diet-induced hepatic TLR4 expression. (a) Representative western blot of TLR4 and GAPDH. (b) Quantification of TLR4 expression to GAPDH expression. SWT, Si-Wu-Tang; *n* = 4-5, ^*∗∗*^*p* < 0.01 vs. Control; ^##^*p* < 0.01 vs. MCD.

**Figure 4:**
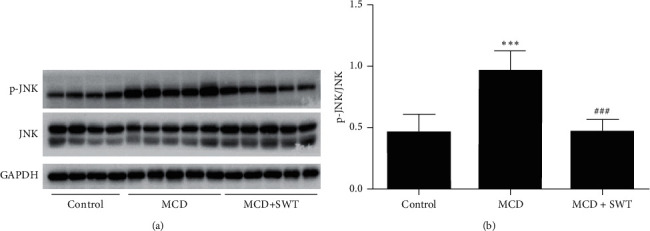
Si-Wu-Tang inhibited MCD diet-mediated JNK activation in liver. (a) Representative western blot of p-JNK, JNK, and GAPDH. (b) Quantification of p-JNK expression to JNK expression. SWT, Si-Wu-Tang; *n* = 4-5, ^*∗∗∗*^*p* < 0.001 vs. Control; ^###^*p* < 0.001 vs. MCD.

Immune/inflammatory dysfunctions play important roles in the pathogenesis of NAFLD. Based on the anti-inflammatory effects of Si-Wu-Tang, therefore, we investigated that whether the protective effects of Si-Wu-Tang on NAFLD were related to TLR4 expression. TLR4 was increased in the liver of mice fed with a MCD diet, while it was reduced by two weeks of Si-Wu-Tang treatment (Figures 3(a) and 3(b)). Subsequently, we examined JNK activation in liver. MCD diet increased hepatic JNK phosphorylation, while JNK activation in liver was inhibited by Si-Wu-Tang (Figures 4(a) and 4(b)).

### 3.5. Si-Wu-Tang Suppressed Hepatic Caspase-8-GSDMD Signaling in Mice Fed with a MCD Diet

**Figure 5:**
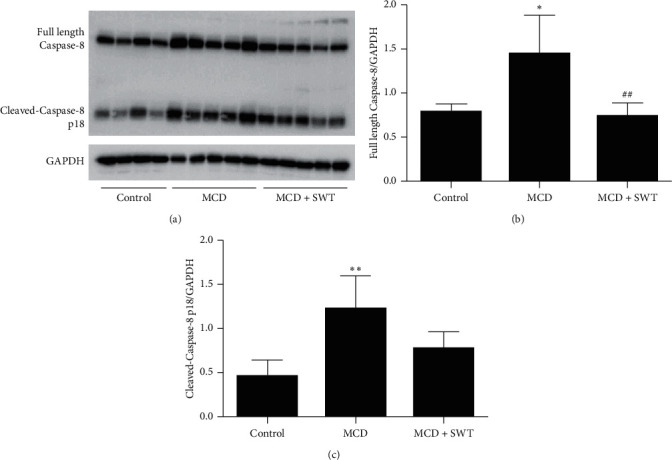
Si-Wu-Tang modulated MCD diet-induced hepatic Caspase-8 expression and activation. (a) Representative western blot of full length Caspase-8, cleaved-Caspase-8 p18, and GAPDH. (b) Quantification of full length Caspase-8 expression to GAPDH expression. (c) Quantification of cleaved-Caspase-8 p18 expression to GAPDH expression. SWT, Si-Wu-Tang; *n* = 4-5, ^*∗*^*p* < 0.05 vs. Control; ^*∗∗*^*p* < 0.01 vs. Control; ^##^*p* < 0.01 vs. MCD.

**Figure 6:**
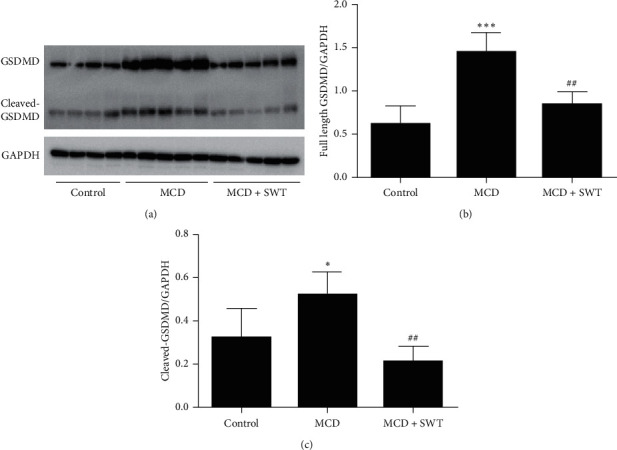
Si-Wu-Tang reduced MCD diet-induced hepatic GSDMD expression and activation. (a) Representative western blot of GSDMD, cleaved-GSDMD, and GAPDH. (b) Quantification of GSDMD expression to GAPDH expression. (c) Quantification of cleaved-GSDMD expression to GAPDH expression. SWT, Si-Wu-Tang; *n* = 4-5, ^*∗*^*p* < 0.05 vs. Control; ^*∗∗∗*^*p* < 0.001 vs. Control; ^##^*p* < 0.01 vs. MCD.

As we have mentioned above, the Caspase-8-GSDMD signaling pathway is essential in the pathogenesis of NAFLD/NASH. Therefore, we investigated whether Si-Wu-Tang could modulate Caspase-8-GSDMD signaling in liver. As shown in Figure 5, the expressions of hepatic full length Caspase-8 and the Caspase-8 active fragment p18 were increased in mice fed with a MCD diet; Si-Wu-Tang suppressed the expression of full length Caspase-8 in liver and decreased the Caspase-8 active fragment p18 levels (with no statistical significance). Moreover, the expression of GSDMD and cleaved-GSDMD were increased in liver of mice fed with a MCD diet; all these were inhibited by Si-Wu-Tang treatment (Figure 6).

## 4. Discussion

The high global or nationwide prevalence of NAFLD underscores the urgent need for effective and safe therapy. However, there are no medications approved by the U.S. Food and Drug Administration (FDA) or European Medicines Agency for the treatment of NAFLD or NASH [78]. Here, we reported that Si-Wu-Tang has the therapeutic potential for NAFLD.

The MCD diet-induced NAFLD animal model is very reproducible, in which animals rapidly develop the clinical pathologies from macrovesicular steatosis to hepatic fibrosis [76]. Mechanistically, choline or methionine stimulates the synthesis of phosphatidylcholine and increases the cellular phosphatidylcholine levels, which is required for the secretion of very-low-density lipoprotein (VLDL) and its deficiency induces lipid accumulation in the liver [76, 79]. The methionine-deficient diet induces mitochondrial S-adenosyl-L-methionine (SAM) and glutathione (GSH) depletion due to perturbing mitochondrial membrane dynamics associated with decreased phosphatidylcholine/phosphatidylethanolamine ratio [76, 80]. Moreover, MCD diet impairs mitochondrial *β*-oxidation and induces cytochrome P450 2E1 (CYP2E1) expression; ROS produced by CYP2E1 *ω*-oxidation, coupled with the depletion of hepatic antioxidants (e.g., reduced SAM and GSH), amplify oxidative damage, thus inducing steatohepatitis [81–85]. In this study, we established the MCD diet-induced NAFLD mice model and found that two weeks of Si-Wu-Tang treatment alleviated MCD diet-induced elevated serum ALT and AST levels and improved liver steatosis, supporting the hepatic protective effects of Si-Wu-Tang.

TLR4 innate immune axis plays an essential role in the pathogenesis of NAFLD, and modulating TLR4 expression and its related signaling pathways have the beneficial effect on NAFLD [32–36, 86, 87]. Genetically, TLR4 deficiency improved NAFLD in mice models [32–34]. Transmembrane BAX inhibitor motif-containing 1 (TMBIM1), which is a multivesicular body (MVB) regulator, protected against NAFLD in mice and monkeys by targeting the lysosomal degradation of TLR4 [87]. In this study, the increased expression of hepatic TLR4 in NAFLD mice was inhibited by Si-Wu-Tang. Besides LPS, the release of high-mobility group box1 (HMGB1) from hepatocytes can also combine with TLR4, thus contributing to the development of NAFLD by inducing JNK activation [34, 37, 38]. Si-Wu-Tang reduced MCD diet-induced hepatic JNK phosphorylation. Therefore, Si-Wu-Tang alleviated MCD diet-induced NAFLD in part via inhibiting TLR4-JNK signaling.

Stimulation of TLR4 or JNK can also induce Caspase-8 activation [40–48]. Caspase-8 activation is the characteristic of murine and human alcoholic liver disease (ALD), deficiency of Caspase-8 in hepatocyte (Casp8^Δhepatocyte^) alleviated steatosis and reduced hepatic triglyceride and free fatty acid (FFA) content in ALD mice model [50]. Moreover, MCD feeding triggered steatosis, hepatic lipid storage, and accumulation of FFA in wild-type livers, which were significantly reduced in Casp8^Δhepatocyte^ animals [39]. Therefore, hepatocyte Caspase-8 is critical for the pathogenesis of steatohepatitis, drugs targeting Caspase-8 might be a plausible treatment for NAFLD/NASH [39]. In our study, the MCD diet-triggered hepatic Caspase-8 expression was inhibited by two weeks of Si-Wu-Tang treatment, which indicated that Caspase-8 is a drug target for Si-Wu-Tang in treating NAFLD.

Recently, two independent studies indicated that Caspase-8 activation mediated by TLR4 resulted in cleavage of GSDMD, leading to pyroptosis [51, 52]. GSDMD and its pyroptosis-inducing fragment GSDMD-N were upregulated in liver tissues of human NAFLD/NASH; MCD diet-fed GSDMD^−/−^ mice exhibited decreased severity of steatosis and inflammation compared with wild type littermates [55]. In our study, the expression of hepatic GSDMD and cleaved-GSDMD were increased in the NAFLD mice model induced by feeding with a MCD diet. The elevated hepatic GSDMD and cleaved-GSDMD levels were inhibited by Si-Wu-Tang, which indicated that Si-Wu-Tang can target GSDMD to alleviate NAFLD. Therefore, the protective effect of Si-Wu-Tang on NAFLD was involved in suppressing Caspase-8-GSDMD signaling.

Our study confirmed the therapeutic effect of Si-Wu-Tang on MCD diet-induced NAFLD in mice in part via modulating innate immune signaling. However, we only analyzed the TLR4-JNK signaling and Caspase-8-GSDMD signaling pathways; other essential innate immune signaling pathways involved in NAFLD, the expression of inflammatory cytokines, and the key genes involved in hepatic lipids metabolism are not assessed.

## 5. Conclusions

Our present study indicated that Si-Wu-Tang alleviates MCD diet-induced NAFLD in mice in part via inhibiting TLR4-JNK signaling and Caspase-8-GSDMD signaling. In conclusion, Si-Wu-Tang may have potential for clinical application in treating NAFLD.

## Figures and Tables

**Table 1:** The formula of Si-Wu-Tang (according to *Tai Ping Hui Min He Ji Ju Fang* (the official Chinese medicine classics in China Song Dynasty) and references [17] and [20]).

Chinese name	Scientific name (family)	Place of origin	Harvest season	Weight (g)
Shu Di Huang (熟地黄, *Rehmanniae*)	Root of *Rehmannia glutinosa* Libosch (Scrophulariaceae)	Henan, China	Autumn	15
Dang Gui (当归, *Angelicae*)	Root of *Angelica sinensis* Diels (Umbelliferae)	Gansu, China	Late Autumn	10
Bai Shao Yao (白芍药, *Paeoniae*)	Root of *Paeonia lactiflora* Pall (Paeoniaceae)	Anhui, China	Summer	10
Chuan Xiong (川芎, *Chuanxiong*)	Rhizome of *Ligusticum chuanxiong* Hortorum (Umbelliferae)	Sichuan, China	Summer	6

**Table 2:** The body weight of mice at baseline and after treatment with SWT.

Group	Body weight (0 week)	Body weight (4th week)
Control	24.70 ± 0.82	26.03 ± 0.89^*∗∗∗*^
MCD	25.00 ± 0.82	16.97 ± 0.62
MCD + SWT	24.90 ± 0.57	18.64 ± 0.38^###^

SWT, Si-Wu-Tang; *n* = 10, ^*∗∗∗*^*p* < 0.001 vs. MCD or MCD + SWT; ^###^*p* < 0.001 vs. MCD.

## Data Availability

The data used to support the findings of this study are included within the article.
